# Childhood and Adolescence Cancers in the Palermo Province (Southern Italy): Ten Years (2003–2012) of Epidemiological Surveillance

**DOI:** 10.3390/ijerph15071344

**Published:** 2018-06-26

**Authors:** Walter Mazzucco, Rosanna Cusimano, Sergio Mazzola, Giuseppa Rudisi, Maurizio Zarcone, Claudia Marotta, Giorgio Graziano, Paolo D’Angelo, Francesco Vitale

**Affiliations:** 1 Department of Science for Health Promotion and Mother to Child Care “G. D’Alessandro”, University of Palermo, via del Vespro, 133 Palermo, Italy; walter.mazzucco@unipa.it (W.M.); giorgio.graziano@gmail.com (G.G.); francesco.vitale@unipa.it (F.V.); 2 Clinical Epidemiology and Cancer Registry Unit, “P. Giaccone” University Hospital, via del Vespro, 133 Palermo, Italy; mazzolasergio3@gmail.com (S.M.); zarcone7@gmail.com (M.Z.); 3 Local Health Unit 6, via Giacomo Cusmano, 24 Palermo, Italy; rosanna.cusimano.55@gmail.com (R.C.); grudisi@libero.it (G.R.); 4 Paediatric Haematology and Oncology Unit, ARNAS “Civico—Di Cristina—Benfratelli”, Piazza Nicola Leotta, 4 Palermo, Italy; oncoematoped@arnascivico.it

**Keywords:** cancer in childhood and adolescence, population-based cancer registries, epidemiological surveillance, cancer incidence, cancer survival, jointpoint regression, time trends

## Abstract

Italy has one of the highest paediatric cancer incidence rates in Europe. We compared cancer incidence and survival rates in children (0–14 years) and adolescents (15–19 years) residing in Palermo Province (PP) with statistics derived from Italian and European surveillance systems. We included all incident cancer cases, malignant tumours and non-malignant neoplasm of central nervous system (benign and uncertain whether malignant or benign), detected in children and adolescents by the Palermo Province Cancer Registry (PPCR) between 2003 and 2012. A jointpoint regression model was applied. Annual Average Percentage Changes were calculated. The Besag–York-Mollie model was used to detect any cluster. The 5-year survival analysis was computed using Kaplan-Meier and actuarial methods. We identified 555 paediatric cancer incident cases (90% “malignant tumours”). No difference in incidence rates was highlighted between PPCR and Italy 26 registries and between PPCR and Southern Europe. No jointpoint or significant trend was identified and no cluster was detected. The 5-year overall survival didn’t differ between PP and the Italian AIRTUM pool. A borderline higher statistically significant survival was observed in age-group 1–4 when comparing PPCR to EUROCARE-5. The epidemiological surveillance documented in the PP was a paediatric cancer burden in line with Italy and southern Europe. The study supports the supplementary role of general population-based cancer registries to provide paediatric cancer surveillance of local communities.

## 1. Introduction

Even if childhood cancer is a rare event [1], it is one of the major causes of death in the younger ages of life [2]. Childhood cancer has a worldwide impact of about 100,000 deaths every year before the age of 15, more than 90% of which occur in resource-limited countries [3,4]. Despite important therapeutic progress and improvement in survival statistics [5,6,7,8], paediatric (0–19) cancer is still a major global health issue with a strong ethical impact [9].

Recent data provided by the third volume of the International Incidence of Childhood Cancer (IICC-3) highlighted a worldwide variation in the overall incidence of childhood cancers by geographical region; the southern European region being the one with the highest rates at 0–14 (world age-standardised rate: 170.9 per million) and 15–19 (world age-standardised rate: 243.7 per million) age groups [10]. Also, a clear increase in the overall rates of childhood neoplasm in the period between the 1980s and the 2000s has been documented [11,12]. 

In Italy, a significant increase in malignant cancer incidences was observed in children (0–14 years) until 1997, with an Annual Percentage Change (APC) of +3.2%, followed by a plateau, while a significant increase in incidence rate was observed in adolescents (15–19 years) between 1998 and 2008 [13]. Data collected by the Italian Network of Cancer Registries (AIRTUM), with regard to the 26 Italian local cancer registries over the period between 1992–2013, confirmed Italy as one of the European countries with the highest incidence rates, ranking third place for both the 0–14 and 15–19 age groups, with a standardized incidence rate of 186.1 per million persons-years and 275.4 per million persons-years, respectively [12].

As reported by the population-based study Eurocare-5, providing survival estimates from 113 European cancer registries, between 2000 and 2007 the 5-year overall survival for childhood cancers in Europe has raised to 77.9% (95%CI: 77.4–78.3%). However, despite the documented improvements in survival, disparities between European countries are still observed, with the lowest outcomes detected in Eastern Europe [14].

In Italy, in the last 15 years, malignant neoplasms occurring in the age-group 0–14 years have shown a consistent 5-year survival improvement with a value of 82% documented in the period between 2003–2008, while a 5-year survival of 86% was reported in adolescents aged 15–19 [13]. 

Furthermore, in Italy, no significant geographical heterogeneity in childhood cancer incidence and survival rates was documented for any type of neoplasms nor for site groups, which is in contrast to cancers that occurred in adults [13,15,16]. 

When compared to the adult population, paediatric patients are affected by different patterns of cancer with peculiar biological and clinical aspects, including both typical childhood tumours and other adolescent epithelial cancers mostly occurring in adults [11,17]. Leukaemia, central nervous system (CNS) neoplasm and lymphoma are the most frequently diagnosed tumour types occurring in children aged 0–14, while lymphomas and epithelial cancers are the most represented in the 15–19 age group [1,7]. 

Despite the fact that aetiology of childhood cancer remains unclear, a possibly infectious aetiology has been postulated [18], while genetic predisposition and environmental factors have been investigated [19], being preconception, *in-utero* or postnatal environmental exposures, which are all potentially implicated [20]. Therefore, childhood cancers have been investigated by cross-space-time clustering analyses [21,22], emphasizing the importance of the surveillance activity performed by population-based cancer registries, also in response to a specific increase in the demand for epidemiological information coming from clinicians and local communities. In Italy, to date only three specialised child and adolescent cancer registries are operative [23], while general population-based cancer registries, covering any age-group including 0–19, are in charge of cancer surveillance for about 70% of the resident population [24].

Since 2003, the Palermo Province Cancer Registry (PPCR) provides the epidemiological cancer surveillance of the fifth most populated area of Italy, with about 1,250,000 residential inhabitants, including a 0–19 year old sub-population of 268,727 subjects (139,811 residing in the Palermo metropolitan area only and the remaining residents in the surrounding 81 municipalities) [25].

In order to investigate the role of the general, population-based cancer registries (GPCRs) with regard to paediatric cancer surveillance, we analysed data of cancer incidences and survival during a ten-year period in child (0–14 years) and adolescent (15–19 years) residents in the Palermo Province (PP). A further comparison of cancer’s epidemiological impact on childhood and adolescence between PP and data derived from the Italian and European surveillance systems was performed.

## 2. Materials and Methods

According to standards and guidelines for cancer registration in Europe provided by European Network cancer registries [26] and the International Agency for Research on Cancer (IARC) [27], we have selected all incidents of tumour cases (“all tumours”), including malignant tumours, as defined according to the fifth digit of the morphology code (behaviour/3), and non-malignant neoplasm of the central nervous system (benign and uncertain whether malignant or benign), coded by using the ICD-O classification III edition (ICD-O-3) [28], which occurred between 2003 and 2012 in resident children (0–14 years-old) and adolescents (15–19 years-old) in the PP. Then, all tumour cases were manually trans-coded by using the third revision of International Classification of Childhood Cancer (ICCC-3) [29]. 

Furthermore, according to the recommendations for a standard dataset for the European Network of Cancer Registries [26], we have explored the following quality indicators of registration: (a) percentage of microscopic verification (MV%): diagnosis based on cytology and/or histology of a metastasis and/or histology of a primary tumour; (b) percentage of Death Certificate Only (DCO%): diagnosis made from death certificate (no information available except the cause and the date of death); c) percentage of non-microscopic verification (NMV%): diagnosis made before death and based only on clinical-instrumental investigation, except histology. There was no case with an “unknown” basis of diagnosis. Cases microscopically verified ranged between 81.8% and 92.3% for “all tumours” (Table 1a) and between 86.7% and 94.5% for “malignant tumours” (Table 1b), respectively, while only one malignant case was reported as DCO. 

We have identified n. 555 neoplasms, n. 500 of which were “malignant tumours” (Table 1). The tumour cases were further categorized in the 12 tumours’ main groups (Table 2). 

Absolute and relative frequencies, and male to female ratio when appropriate, were calculated. The cancer incidence rate per million persons-years was standardized on European population (Std-EU) and was computed for the 0–14 age group using the following weights per each age group: 0–4 = 0.8, 5–9 = 0.7, 10–14 = 0.7. Age-specific incidence rates per million persons-years were calculated for the 15-19 age group per year each year within the study period [13]. The rates were calculated using the annual inter-census estimates between 2002–2012 of the resident population, males and females aged 0–19 and by a single year of age, provided by the Italian Statistics Institute (ISTAT) [25]. Further, a comparison between age-specific incidence rates (0, 1–4, 5–9, 10–14, 15–19) in the PP and in the pool of the 26 Italian registries providing childhood cancer data for the period between 1992–2013 (26 Italian registries) [10] was performed using Fisher’s test with a statistical significance set at *p*-value = 0.05. Moreover, the comparison of cancer incidence in children and adolescents between PPCR and the “southern European region” was performed using world standardized population rates (WSR) [11]. 

The time trends of cancer incidence rates were fitted under a log-linear model by using the Jointpoint Regression Program software [30]. The jointpoint regression model was applied to identify any modification in time trends for Std-EU rates in the 0–14 age-groups and age-specific incidence rates for the 15–19 age-group. The permutation test obtained by the grid search method suggested by Lerman [31] was used in order to determine the number of significant jointpoints between 2003–2012. Given the size of the available time series, the maximum detectable jointpoints was equal to 1. Significance was set at α = 0.05 and the test was adjusted by using the Bonferroni correction. Any differences in Annual Percentage Changes (APCs) between the two time periods in consideration were calculated.

Annual Average Percentage Changes (AAPCs) were calculated by fitting the squares’ regression line to the natural logarithm of the rates. When the regression coefficient β is equal to zero then AAPC equals zero.

The incident cancer cases were localized using a space-time combination of address and date of diagnosis [32]. Standardized incidence ratio (SIR) for the ten-year study period was calculated by indirect method for each PP municipality. To perform the spatial analysis, SIRs were corrected using Besag-York-Mollie (BYM) Bayesian method [33]. SIR-BYM and the post-probability (PProb) maps were generated. PProb higher than 0.95 was considered statistically significant.

The 5-year survival analysis was restricted to “malignant tumours” only. For multiple tumours we followed the IARC-IACR rules [27]. End of follow-up was set at 31 December 2017. Cases lost to follow-up were 0.8%. 

The 5-year observed survival and 95% Confidence Intervals (CIs) in the age groups 0–14 and 15–19 were computed applying the Kaplan-Meier method to compare the results with AIRTUM pool, available only for the time period between 2003–2008 [14], while the 5-year observed survival and 95%CIs in the age groups <1, 1–4, 5–9 and 10–14 were calculated by using the actuarial method to make comparisons with the Eurocare-5 study, available only for the period between 2000–2007 [14]. The computed 95%CIs were used to visually compare the survival data observed in the PP with the selected published data [13,14]. 

BYM estimation, cluster analysis and survival analysis were performed by using RStudio (version 0.98.945, RStudio Inc., Boston, MA, USA) for the software R (3.1.—2014-04-10, R Foundation for Statistical Computing, Vienna, Austria) with packages INLA, DCluster and surv, respectively.

The study was conducted in accordance with the Declaration of Helsinki, and the protocol was approved by the Ethics Committee of Palermo “Palermo 1“ (N. 05/2017).

## 3. Results

We identified 555 incidents of cancer cases (312 males and 243 females), defined as “all tumours” including malignant tumours and non-malignant neoplasm of the central nervous system (benign and uncertain whether malignant or benign), coded by using the ICD-O-3 [21], and registered in children and adolescents by the PPCR between 2003 and 2012 (Table 1a). Within the study cohort, 500 cases (279 males and 221 females) were “malignant tumours” (Table 1b). Moreover, the distribution of incident cases was comparable for genders and age-groups in both “all tumours” and “malignant tumours” categories, the observed frequency being higher in males than in females and the male to female ratio being higher than 1 in all age groups. 

Table 2 reports the frequency distribution of the incidents of cancer cases according to the 12 main ICCC-3 groups for both 0–14 and 15–19 age categories. Within the age class, 0–14 Leukaemia was the most frequent (28%), followed by CNS neoplasm (25%), Lymphoma & related (11%), Neuroblastoma (10%), Soft tissue sarcoma (6%), Renal and Bone tumours (5% respectively), Germ cell and Carcinoma & Melanoma (3% respectively), Others & unspecified tumours and Retinoblastoma (2% respectively), and Hepatic tumours (1%).

Within the age-class 15–19 Lymphoma and related were the most represented (31%), followed by Carcinoma & Melanoma (22%), Leukaemia (14%), CNS neoplasm (12%), Germ cell tumours (8%), and Bone tumours and Soft tissue sarcoma (6% respectively), Renal and Others & unspecified (1% respectively).

**Figure 1:**
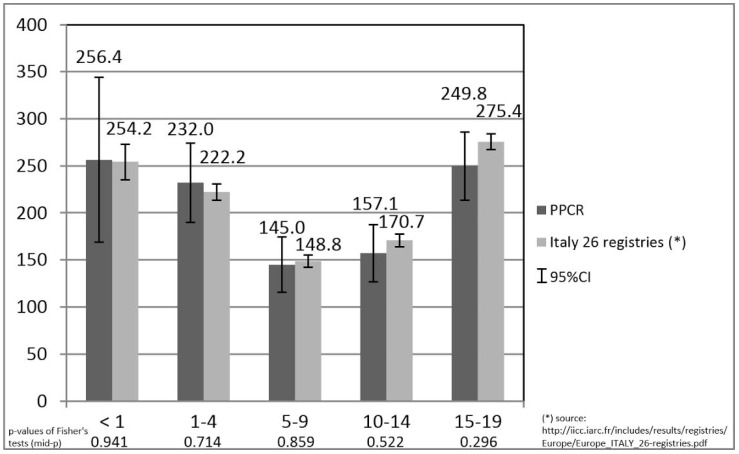
Age-specific incidence rates of paediatric tumours (males and females together): comparison between Palermo Province Cancer Registry (2003–2012) and “Italy 26 registries” (1992–2013). **Figure 1 legend.** PPCR: Palermo Province Cancer Registry; 95%CI: 95% Confidence Intervals.

Figure 1 depicts the comparison between PPCR and “Italy 26 registries” for “all tumours” age-specific incidence rates: there was no statistical difference (*p*-value > 0.05) in all of the considered age-groups (<1, 1–4, 5-9, 10–14 and 15–19).

There was no difference when comparing incidence rates between PPCR (Std-EU = 182.3; 95%CI: 163.3–201.2) and “Italy 26 registries” (Std-EU = 180.0; 95%CI: 174.0–186.0) for the 0–14 age-group. No difference in children was reported when we compared incidences between PPCR (WSR = 185.6; 95%CI: 166.3–204.9) and “Southern European Region” (WSR = 170.9). In the same direction, incidences of cancer in adolescents has not shown any difference between PPCR (ASR = 249.8; 95%CI: 214.7–284.8), “Italy 26 registries” (ASR = 275.4; 95%CI: 262.4–288.4) and “Southern European Region” (ASR = 243.7) (data not shown).

**Figure 2:**
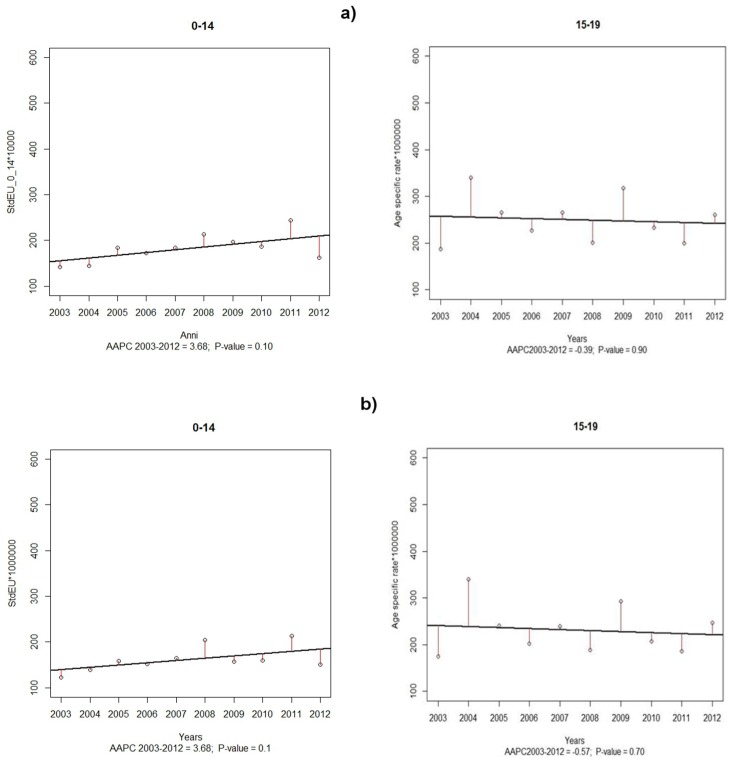
Average Annual Percentage Changes for (**a**) “all tumours” (males and females together) and (**b**) “malignant tumours”, by age-groups. Palermo Province, 2003–2012. **Figure 2 legend.** AAPC: Annual Average Percentage Change.

Figure 2 represents the results of the jointpoint regression model and the values of the AAPCs for both “all tumours” (a) and “malignant cancers only” (b) in the study period. No jointpoint has been identified in incidence time-trends for childhood and adolescent cancer groups for “all tumours” nor for “malignant cancers only”. 

Further, no statistically significant trend was detected in any considered age-group, neither for “all tumours” nor “malignant tumours”. 

**Figure 3:**
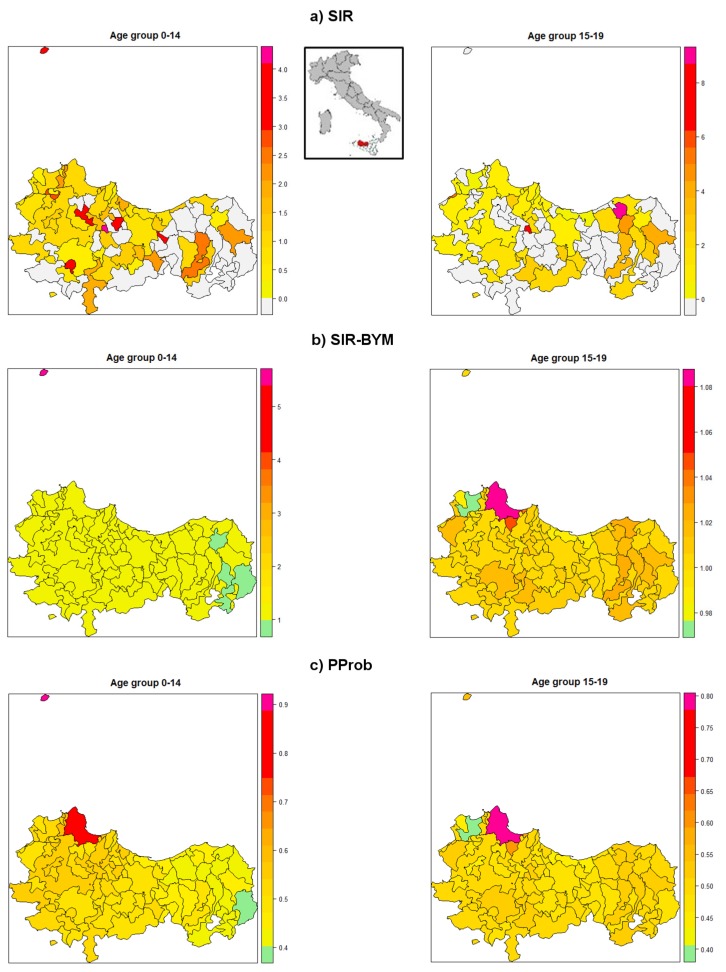
Spatial analysis of (**a**) SIRs of “all tumours”; (**b**) BYM estimates and their (**c**) post-probabilities by municipality and by children and adolescent cancers. Palermo Province (males and females together), 2003–2012. SIR: Standardized Incidence Ratio; SIR-BYM: estimates of SIRs from Besag-York-Mollie Bayesian model; PProb: post-probabilities of BYM models.

Figure 3 shows the SIRs by age-group and by municipality of (a) “all tumours”, the (b) BYM estimates and their (c) Pprobs. Despite the significance of many municipality SIRs (results not shown), as the maximum Pprob value was of 88%, no significant BYM estimate was highlighted. Therefore, no cluster was identified. 

Table 3 shows the comparison of the 5-year survival rate restricted to “malignant tumours” between PPCR and Italian AIRTUM pool [13] and between PPCR and EUROCARE-5 study [12]. A 5-year overall survival rate of 82% (95%CI: 78–87) for the childhood group and of 86% (95%CI: 81–91) for the adolescent group was documented in the PP during the study period, but no statistically significant difference was reported with regard to the Italian AIRTUM pool (Table 3a). 

A 5-year survival of 85% (95%CI: 74–98) for the <1 age-group, of 86% (95%CI: 80–93) for the 1–4 age-group, of 84% (95%CI: 76–93) for the 5–9 age-group and of 85% (95%CI: 78–93) for the 10–14 age-group was documented in the PP, with a borderline higher statistically significant survival in the age-group 1–4 when comparing PPCR to EUROCARE-5 (Table 3b). 

## 4. Discussion

This study reports incidences and survival statistics of tumour cases which occurred in childhood (0–14 years) and adolescence (15–19 years) over a ten-year period in the PP. Our results summarize the epidemiological impact of childhood and adolescence tumours on the fifth Italian administrative most populated area within a country documenting one of the highest incidences of cancer rates in Europe [13], allowing us at the same time to make comparisons between both national and European frameworks, particularly the southern European region, by using data derived from a general population-based cancer registry. 

We preferred to analyse data considering the 0–14 and 15–19 age categories, instead of the commonly used 0–14 and 0–19 age-groups, according to the different characteristics of the tumours occurring within the two chosen age-groups (distribution of neoplasm with heterogeneous prognosis, treatment protocols and diagnostic-therapeutic strategies and host biology) [14] and the well-known difficulties of adolescents to be diagnosed and properly cured. This choice was also related to the availability of data deriving from a GPCR: as its registration rules, unlike paediatric-specialized cancer registries, are not exclusively designed to collect data on paediatric cancers [34,35], GPCRs codify childhood and adolescence incident tumour cases by using the third revision of the ICD-O-3 [28] needing to trans-codify into ICCC-3 [29]. 

Cancers in childhood and adolescence in the PP were more frequent in males than females both for “all tumours” and “malignant tumours”, as expected according to literature [36,37].

Uniformity with national statistics has been highlighted with regard to the relative frequency of tumour types and the burden of disease. In fact, according to the 12 main ICCC-3 groups, the results provided by PPCR showed the same ranking reported by literature [10], the Leukaemia group being the most represented in the 0–14 age category, followed by CNS neoplasm and Lymphoma & related neoplasm groups, while Lymphoma & related neoplasm groups ranked first followed by Carcinoma & Melanoma and Leukaemia groups in the 15–19 age category. 

Further, as compared to “Italy 26 registries” [13], the incidence rates per million persons-years in specific age classes <1, 1–4, 5–9, 10–14 and 15–19 reported by the PPCR resulted in a substantial overlapping. Also, the graphical representation of the age-specific incidence rates showed the classical “U” shape documented by literature [36,37]. 

In the same direction, there was no difference in standardised incidence rates for all the considered age-groups when comparing statistics provided by PPCR to “Italy 26 registries” or to data available for the southern European region, depicting a local and national epidemiological context in line with a wider area documenting the highest incidence rates in children and adolescents as compared to the entire European region where, in the 1990s, an age-standardized cancer incidence rate of 130 per million persons-years was documented among children aged between 0 to 14 years old, whilst, over the period between 1995–2002, the annual overall incidence rate in adolescents (15–19 years) was of 157 per million persons-years [10,12]. 

Nevertheless, the evidences provided by jointpoint regression model and spatial analysis, taken together, make us confident to state that—despite the relatively short observational time window available - the surveillance system documented the absence of environmental risk areas as well as the occurrence of any specifically relevant event in the PP during the study period. 

Within the study limitations, the documented evidences of an improvement over time in registration quality and population coverage of cancer registries [11,38], particularly, immediately after the start-up period, should be taken into account when interpreting our data. 

Moreover, despite the fact that clustering for childhood tumours has been reported in literature, particularly for leukaemia, intracranial and intraspinal embryonal tumours, and Hodgkin lymphomas [18,39,40], the scanty number of specific types of incident cancers registered by the PPCR in a relatively short period made us hesitant to explore clusters for leukaemia or other specific tumour sites. 

The aforementioned limitations together with the documented evidence of an increase in the overall rates of childhood neoplasm in the last decay [11,12] are crucial points to consider, since GPCRs are currently involved in the communication of risk related to environment and cancers and their mission includes the translation and dissemination of evidence to enable informed decision-making and to empower the general population or other stakeholders, while at the same time preserving a rigorous methodological approach [41]. Therefore, according to the available body of evidence, greater attention should be paid with regard to childhood and adolescence tumours in order to explore the implementation of a complementary methodological approach to the commonly used graphical and visual formats (choropleth maps, tables, etc.) also in the risk communication of cancer paediatric burden to stakeholders, particularly to communities and local authorities [41]. 

Lastly, the comparison of the 5-year overall survival rate, restricted to “malignant tumours” only, between data provided by PPCR and the Italian AIRTUM pool [13] has documented overlapping values of point estimates and their CIs for both children and adolescents. Moreover, the 5-year survival point estimates are higher in the PP when comparing the PPCR data with Eurocare-5 study [14], representing survival data on a European scale, even if only a borderline higher statistically significant survival observed in favour of the PP for age-group 1–4.

The previous findings can be explained by the presence of a high quality network of specialized centers for the treatment of paediatric cancers in Italy, ensuring specific healthcare pathways and protocols; also, in relation to the patients’ migration across the country [13].

## 5. Conclusions

The epidemiological surveillance conducted by the PPCR in the PP has highlighted a burden of childhood cancer in line with the Italian and the southern European frameworks. 

As authors agree with the recent proposal to “move towards pan-European coverage” by implementing the institution of childhood specialized cancer registries on a national level [35], potential perspectives for the future are to intensify the collaboration between PPCR and the European Network for Cancer Research in Children and Adolescents [42], as well as with clinicians, as already documented by the previous joint experience conducted by the Italian Association of Paediatric Haematology and Oncology and the AIRTUM network [13]. In this context, the progressive adoption of the new guidelines and recommendations, on which staging systems should be adopted by population-based cancer registries for the major childhood cancers, will make it easy to perform comparative studies on incidences and other outcomes of interest, particularly survival [43]. Nevertheless, if it seems appropriate to think globally, then it is necessary to act locally in order to answer the health demands of each specific community. To this end, the experience of the PPCR supports a supplementary role of general population-based cancer registries, as compared to paediatric specialized cancer registries, to carry out childhood and adolescence cancer surveillance with the aim to assess and control the impact of cancerous diseases on local communities [44].

## Figures and Tables

**Table 1:** Paediatric incident tumours cases by type, age-group and gender. Palermo Province, 2003–2012.

**(a) All Tumours**							
**Cases in Study**					Quality Indicators *		
**Age Group**	**M *n* (%)**	**F *n* (%)**	**Total *n* (%)**	**M/F**	**MV *n* (%)**	**DCO *n* (%)**	**NMV *n* (%)**
0–14	201 (64.4)	159 (65.4)	360 (64.9)	1.26	313 (86.9)	1 (0.3)	46 (12.8)
0	20 (6.4)	13 (5.4)	33 (5.9)	1.54	28 (84.8)	0 (0.0)	5 (15.2)
1–4	64 (20.5)	55 (22.6)	119 (21.5)	1.16	106 (89.1)	0 (0.0)	13 (10.9)
5–9	57 (18.3)	38 (15.6)	95 (17.1)	1.5	82 (86.3)	0 (0.0)	13 (13.7)
10–14	60 (19.2)	53 (21.8)	113 (20.4)	1.13	97 (85.8)	1 (0.9)	15 (13.3)
15–19	111 (35.6)	84 (34.6)	195 (35.1)	1.32	177 (90.8)	0 (0.0)	18 (9.2)
0–19	312 (100)	243 (100)	555 (100)	1.28	493 (88.8)	1 (0.2)	61 (11.0)
**(b) Malignant Tumours**							
**Cases in Study**					Quality Indicators *		
**Age Group**	**M *n* (%)**	**F *n* (%)**	**Total *n* (%)**	**M/F**	**MV *n* (%)**	**DCO *n* (%)**	**NMV *n* (%)**
0–14	175 (62.7)	144 (65.2)	319 (63.8)	1.21	285 (89.4)	1 (0.3)	33 (10.3)
0	17 (6.1)	13 (5.9)	30 (6.0)	1.31	26 (86.7)	0 (0.0)	4 (13.3)
1–4	57 (20.4)	50 (22.7)	107 (21.4)	1.14	98 (91.6)	0 (0.0)	9 (8.4)
5–9	49 (17.6)	33 (14.9)	82 (16.4)	1.48	72 (87.8)	0 (0.0)	10 (12.2)
10–14	52 (18.6)	48 (21.7)	100 (20.0)	1.08	89 (89.0)	1 (1.0)	10 (10.0)
15–19	104 (37.3)	77 (34.8)	181 (36.2)	1.35	171 (94.5)	0 (0.0)	10 (5.5)
0–19	279 (100)	221 (100)	500 (100)	1.26	456 (91.2)	1 (0.2)	43 (8.6)

M: males; F: females; M/F: ratio between males and females; MV: microscopic verification; DCO: death certificate only; NMV: non-microscopic verification. * There was no case with an “unknown” basis of diagnosis.

**Table 2:** Childhood and adolescence tumours incident cases (*n* 555) by third revision of International Classification of Childhood Cancer (ICCC-3) groups and age-group. Palermo Province, 2003–2012.

ICCC-3 Main Groups	Children (0–14 Age Group)		Adolescents (15–19 Age Group)	
	*n* of Cases	%	*n* of Cases	%
I LEUKEMIA	99	28	27	14
a Lymphoid	73	74	17	63
b Acute myeloid	20	20	9	33
c CMD	-	-	-	-
d MDS & other	3	3	1	4
e Unspecified	3	3	-	-
II LYMPHOMA & RELATED	41	11	60	31
a Hodgkin	21	51	37	62
b Non-Hodgkin except BL	7	17	19	32
c Burkitt (BL)	6	15	1	1.5
d Lymphoreticular	6	15	2	3
e Unspecified	1	2	1	1.5
III CNS NEOPLASMS	91	25	24	12
a Ependymoma	3	3	1	4
b Astrocytoma	31	34	6	25
c CNS embryonal	8	9	5	21
d Other gliomas	7	8	2	8
e Other specified	11	12	5	21
f Unspecified CNS	31	34	5	21
IV NEUROBLASTOMA	37	10	-	-
a (Ganglio) neuriblastoma	35	95	-	-
b Peripheral nervous	2	5	-	-
V RETINOBLASTOMA	6	2	0	0
VI RENAL TUMOURS	19	5	2	1
a Nephroblastoma	17	89	1	50
b Renal carcinoma	2	11	1	50
c Unspecified	-	-	-	-
VII HEPATIC TUMOURS	3	1	1	1
a Hepatoblastoma	3	100	-	-
b Hepatic carcinoma	-	-	100	100
c Unspecified	-	-	-	-
VIII BONE TUMOURS	18	5	12	6
a Oteosarcoma	8	44	1	8
b Chondrosarcoma	-	-	-	-
c Ewing & related	7	39	5	42
d Other specified	-	-	-	-
e Unspecified	3	17	6	50
IX SOFT TISSUE SARCOMA	20	6	11	6
a Rhabdomyosarcoma	10	50	1	9
b Fibrosarcoma	5	25	4	36
c Kaposi sarcoma	-	-	-	-
d Other specified	4	20	6	55
e Unspecified	1	5	-	-
X GERM CELL TUMOURS	10	3	15	8
a CNS germ cell	2	20	10	66
b Other extragonadal	4	40	-	-
c Gonadal germ cell	4	40	3	20
d Gonadal carcinoma	-	-	1	7
e Unspecified gonadal	-	-	1	7
XI CARCINOMA & MELANOMA	9	3	42	22
a Adrenocortical	-	-	1	2
b Thyroid	6	67	27	64
c Nasopharyngel	1	11	3	7
d Melanoma	1	11	3	7
e Skin carcinoma	-	-	4	10
f Other & unspecified	1	11	4	10
XII OTHER & UNSPEFIED	7	2	1	1
a Other specified	-	-	-	-
b Other unspecified	7	100	1	100
TOTAL	360	100	195	100

**Table 3:** Comparison of five-year observed survival (%) and its 95%CI for “malignant tumours”: Palermo Province Cancer Registry versus Italian AIRTUM pool (**a**) and versus Eurocare-5 study (**b**).

**(a)**	**0–14 Years**		**15–19 Years**	
**Observed Survival % (Kaplan-Meier Method)**	**Overall (95%CI)**		**Overall (95%CI)**	
**PPCR** (2003–2012)	82% (78–87)		86% (81–91)	
**AIRTUM POOL** (2003–2008)	82% (80–83)		86% (84–87)	
**(b)**	**Age Groups**			
**Observed survival % (Actuarial Method)**	**<1 (95%CI)**	**1–4 (95%CI)**	**5–9 (95%CI)**	**10–14 (95%CI)**
**PPCR** (2003–2007)	86% (75–99)	86% (80–93)	81% (73–90)	80% (73–88)
**EUROCARE-5** (2000–2007)	78% (76–79)	79% (78–80)	78% (77–79)	77% (76–78)

## Abbreviations

AAPC	Annual Average Percentage Changes
AIRTUM	Italian Network of Cancer Registries
APC	Annual Percentage Change
ASR	age-specific rates
BL	Burkitt Lymphoma
BYM	Besag-York-Mollie
Cis	Confidence Intervals
CMD	Chronic myeloid disorders
CNS	Central Nervous System
EU	European Union
GAM	Geographical Analysis Machine
GPCR	general population-based cancer registries
IARC	International Agency for research on cancer
ICCC	International Classification of Childhood Cancer
ICD-O	International Classification of Diseases for Oncology
IICC	International Incidence of Childhood Cancer
ISTAT	Italian Statistics Institute
MDS	Myelodysplastic syndromes
PP	Palermo Province
PPCR	Palermo Province Cancer Registry
PProb	post-probability
SIR	standardized incidence ratio
Std-EU	Standardized by age on the European population
WSR	World standardized rate
